# Supplementary material for: Mechanistic Study of Triazole Based Aminodiol Derivatives in Leukemic Cells—Crosstalk between Mitochondrial Stress-Involved Apoptosis and Autophagy
Source: Int J Mol Sci. 2020 Apr 2;21(7):2470. doi: 10.3390/ijms21072470 (PMC7177546; doi:10.3390/ijms21072470)
Supplement: Supplementary file 1 [file ijms-21-02470-s001.pdf]

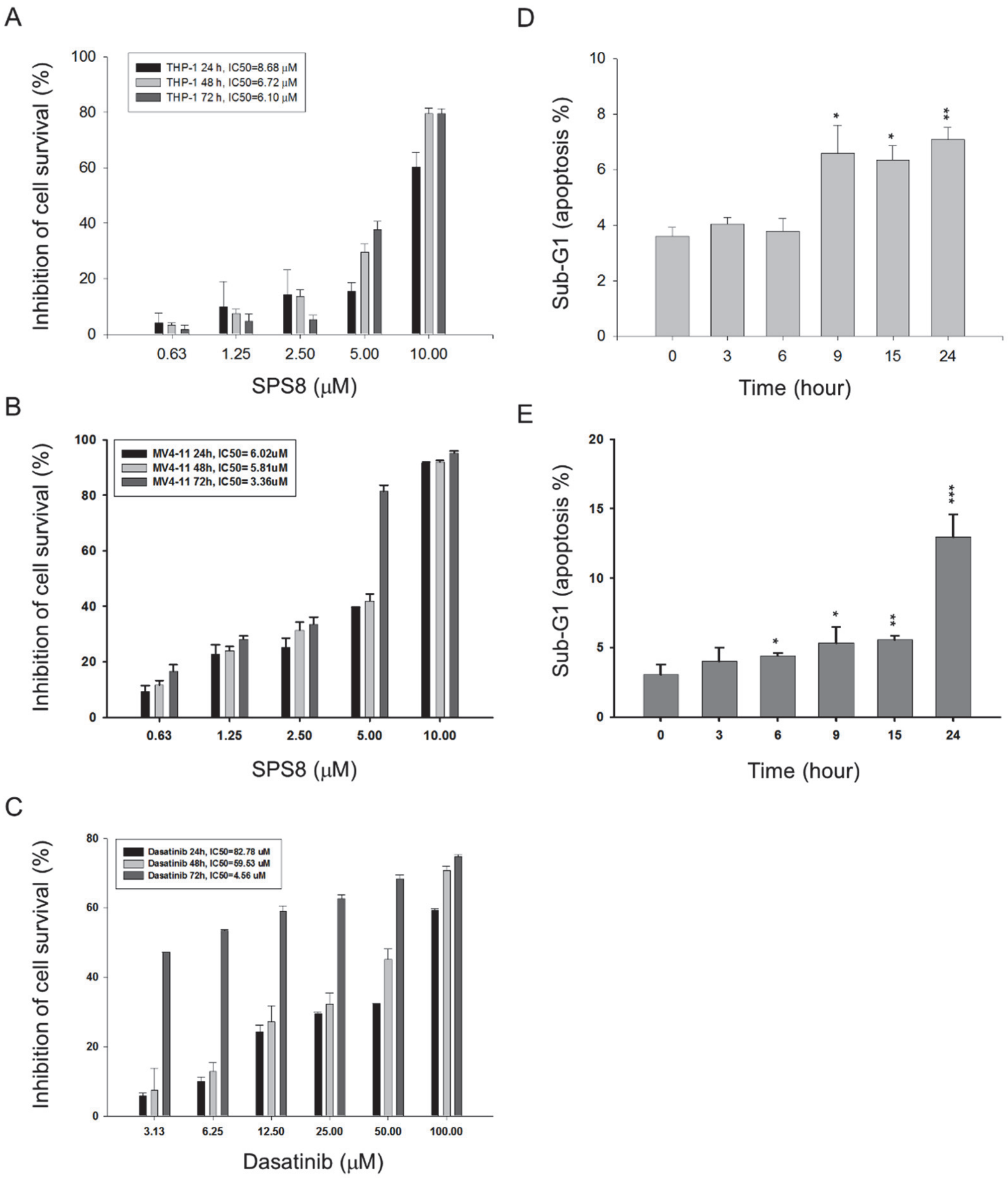

Supplementary Figure S1. Effect of SS8 and dasatinib on cell viability of several leukemic lines. Graded concentrations of SPS8 or dasatinib were added to the cells (A, THP-1; B, MV-4-11; C, HL-60) for 24, 48 or 72 h. The cytotoxic effect was determined by MTT assay. Data are expressed as mean±SEM of three independent experiments. The IC_{50} values were demonstrated at each exposure time. THP-1 (D) and MV-4-11 (E) cells were incubated in the absence or presence of SPS8 (5 μM) for the indicated times. Flow cytometric analysis of DNA staining with propidium iodine was performed. Data are expressed as mean±SEM of three independent experiments. **P* < 0.05, ***P* < 0.01 and ****P* < 0.001 compared with the control.

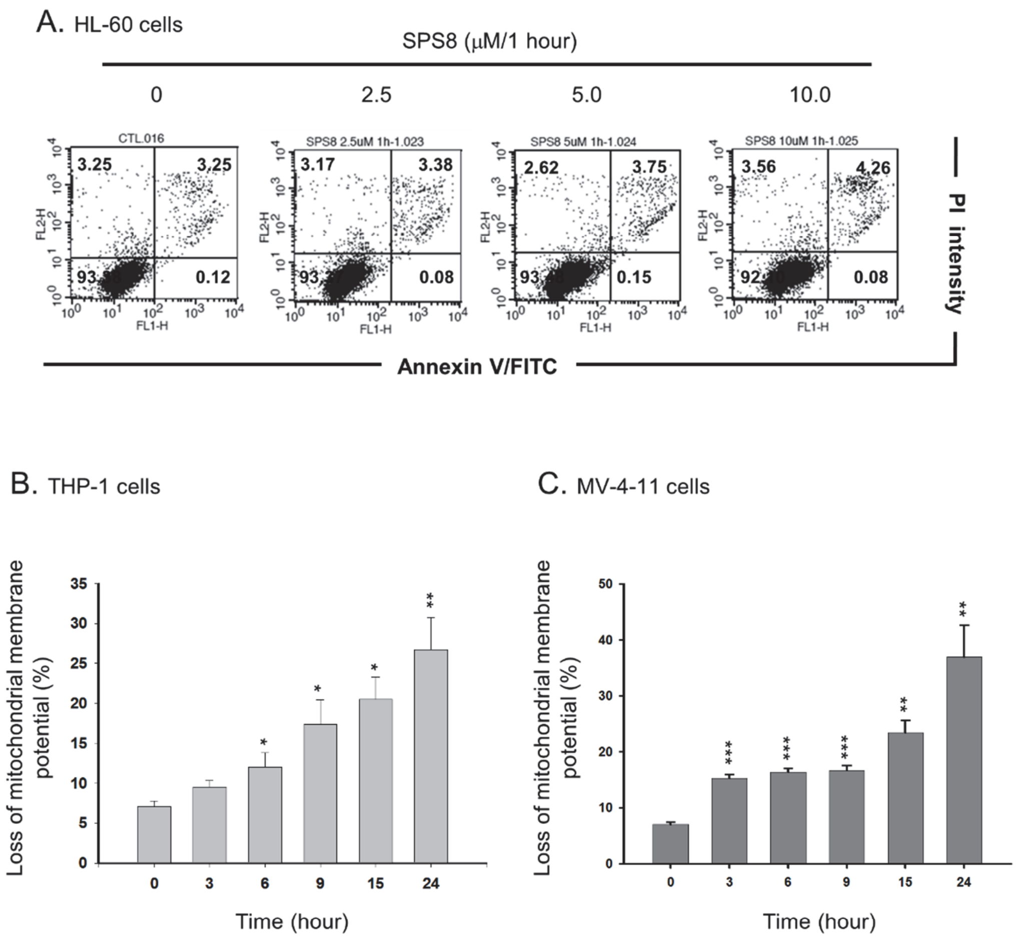

Supplementary Figure S2. Effect of SPS8 on necrosis and mitochondrial membrane potential in several leukemic cell lines. The cells were incubated in the absence or presence of SPS8 (A, 2.5-10 μM; B, 5 μM; C, 5 μM for the indicated times. After the treatment, the cells were incubated with Annexin V-FITC/PI or JC-1 dye for the detection of necrosis or mitochondrial membrane potential, respectively, using flow cytometric analysis. Data are expressed as mean±SEM of three independent experiments. $*P < 0.05$, $**P < 0.01$ and $***P < 0.001$ compared with zero time control.

A

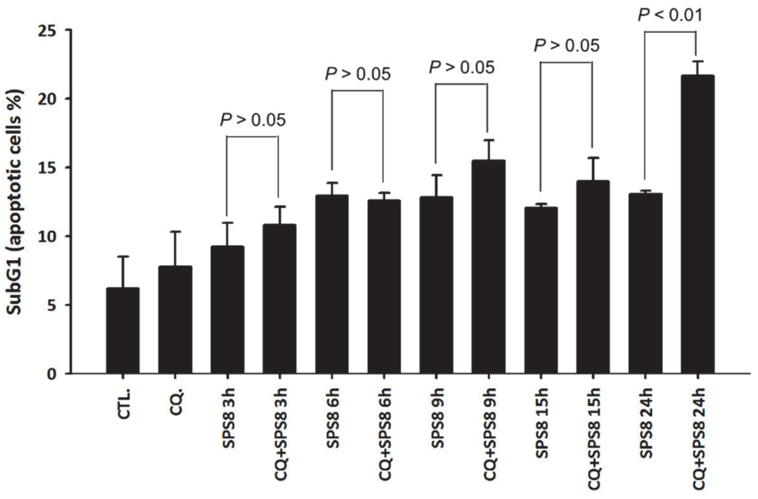

B

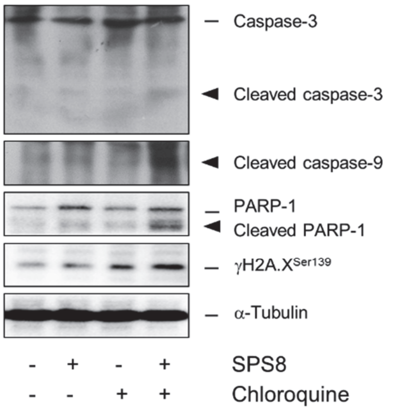

Supplementary Figure S3. Effect of chloroquine on SPS8-induced apoptosis of HL-60 cells. HL-60 cells were incubated in the absence or presence of SPS8 (5 μM) and chloroquine (10 μM) for the indicated times (A) or 24 h (B). Flow cytometric analysis of DNA staining with propidium iodine was performed (A), or the cells were harvested for the detection of protein expression using Western blotting (B). The expressions were quantified using Image Lab Software 6.0 (BIO-RAD). Data are expressed as mean±SEM of three independent experiments.

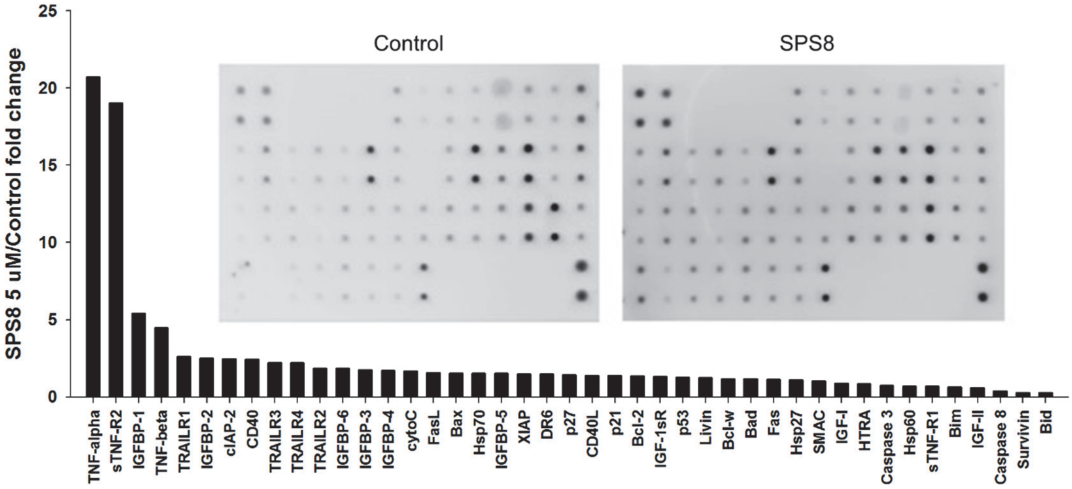

Protein	SPS8/CTL fold change	Protein	SPS8/CTL fold change
TNF-alpha	20.70	CD40L	1.39
sTNF-R2	19.04	p21	1.38
IGFBP-1	5.43	Bcl-2	1.35
TNF-beta	4.46	IGF-1sR	1.33
TRAILR1	2.62	p53	1.29
IGFBP-2	2.49	Livin	1.26
cIAP-2	2.48	Bcl-w	1.17
CD40	2.42	Bad	1.16
TRAILR3	2.22	Fas	1.14
TRAILR4	2.21	Hsp27	1.09
TRAILR2	1.87	SMAC	1.04
IGFBP-6	1.85	IGF-I	0.89
IGFBP-3	1.74	HTRA	0.86
IGFBP-4	1.71	Caspase 3	0.74
cytoC	1.69	Hsp60	0.71
FasL	1.59	sTNF-R1	0.70
Bax	1.54	Bim	0.64
Hsp70	1.52	IGF-II	0.60
IGFBP-5	1.52	Caspase 8	0.39
XIAP	1.50	Survivin	0.28
DR6	1.49	Bid	0.28
p27	1.41		

Supplementary Figure S4. Effect of SPS8 on apoptosis-related protein. Human Apoptosis Antibody Array was performed in HL-60 cells treated with SPS8 (5 μM) or control for 24 h. Forty-three targets detected in the chips with a one-to-one correspondence. The levels of proteins were quantified and data were expressed as fold of control.
